# Simultaneous endovascular repair of an iatrogenic carotid-jugular fistula and a large iliocaval fistula presenting with multiorgan failure: a case report

**DOI:** 10.1186/1752-1947-6-33

**Published:** 2012-01-24

**Authors:** Yuigi Yuminaga, Walid Mohabbat, Tam Nguyen, Andrew Michael Thompson, Charles Marshall Fisher

**Affiliations:** 1 Department of Vascular Surgery, Royal North Shore Hospital Sydney, St Leonards 2065, Australia

## Abstract

**Introduction:**

Iliocaval fistulas can complicate an iliac artery aneurysm. The clinical presentation is classically a triad of hypotension, a pulsatile mass and heart failure. In this instance, following presentation with multiorgan failure, management included the immediate use of an endovascular stent graft on discovery of the fistula.

**Case presentation:**

A 62-year-old Caucasian man presented to our tertiary hospital for management of iatrogenic trauma due to the insertion of a central venous line into his right common carotid artery, causing transient ischemic attack. Our patient presented to a peripheral hospital with fever, nausea, vomiting, acute renal failure, acute hepatic dysfunction and congestive heart failure. A provisional diagnosis of sepsis of unknown origin was made. There was a 6.5 cm×6.5 cm right iliac artery aneurysm present on a non-contrast computed tomography scan. An unexpected intra-operative diagnosis of an iliocaval fistula was made following the successful angiographic removal of the central line to his right common carotid artery. Closure of the iliocaval fistula and repair of the iliac aneurysm using a three-piece endovascular aortic stent graft was then undertaken as part of the same procedure. This was an unexpected presentation of an iliocaval fistula.

**Conclusion:**

Our case demonstrates that endovascular repair of a large iliac artery aneurysm associated with a caval fistula is safe and effective and can be performed at the time of the diagnostic angiography. The presentation of an iliocaval fistula in this case was unusual which made the diagnosis difficult and unexpected at the time of surgery. The benefit of immediate repair, despite hemodynamic instability during anesthesia, is clear. Our patient had two coronary angiograms through his right femoral artery decades ago. Unusual iatrogenic causes of iliocaval fistulas secondary to previous coronary angiograms with wire and/or catheter manipulation should be considered in patients such as ours.

## Introduction

Iliocaval fistulas can complicate an iliac artery aneurysm. The clinical presentation is classically a triad of hypotension, a pulsatile mass and heart failure [1]. In this instance, following presentation with multiorgan failure, management included the immediate use of an endovascular stent graft on discovery of the fistula.

### Case presentation

A 62-year-old Caucasian man was transferred to our tertiary hospital for further management of transient left hemiparesis following iatrogenic trauma due to the insertion of a central line to his right common carotid artery (CCA) at a peripheral center. Our patient had initially presented with symptoms of nausea, vomiting, fever, weakness and dizziness for several days. He did not have any abdominal pain, dypsnoea or urinary symptoms. His background medical history included ischemic heart disease (requiring two previous coronary angiograms with the insertion of a coronary stent to his left anterior descending artery), hypertension, hypercholesterolemia and excessive alcohol consumption (more than 80 g per day). His usual medications were atenolol, felodipine, aspirin and naproxen.

Laboratory studies conducted at the regional hospital showed a creatinine (Cr) level of 269 μmol/L (normal range: 60 μmol/L to 110 μmol/L), aspartate transaminase (AST) of 2309 IU/L (normal range: 8 IU/L to 40 IU/L), alanine transaminase of 1994 IU/L (normal range: 5 IU/L to 60 IU/L), gamma-glutamyltransferase (GGT) of 177 IU/L (normal range: 40 IU/L to 78 IU/L), bilirubin of 33 μmol/L (normal range: 3 μmol/L to 18 μmol/L), white cell count of 27.8 × 10^9^/L (normal range: 4 × 10^9^/L to 11 × 10^9^/L) hemoglobin (Hb) of 120 g/L(normal range: 135 g/L to 180 g/L), an international normalized ratio (INR) of 2.4 and platelet count of 50 × 10^9^/L (normal range: 150 × 10^9^/L to 400 × 10^9^/L). Metabolic acidosis was also noted.

A provisional diagnosis of multiorgan failure due to septic shock was made. Empirical intravenous antibiotics (cefotaxime) were started. A central venous cathether was sited in his right internal jugular vein under ultrasound guidance. Following this, however, he experienced a transient ischemic attack with left-sided weakness lasting five minutes. A duplex ultrasound of his right neck was performed, demonstrating the presence of the central venous catheter (CVC) passing through his right internal jugular vein posteriorly into the lumen of his right CCA. He was subsequently referred to the Vascular Surgery Service at our tertiary hospital for further management.

On admission to our hospital, our patient had a heart rate of 89 beats per minute (atrial fibrillation), blood pressure of 156/56 mmHg and oxygen saturation of 98%. He had mild bilateral leg edema, a palpable liver edge and a raised jugular venous pressure. No pulsatile mass was identified.

In our Intensive Care Unit, a diagnosis of heart failure was made in association with a central venous pressure of 35 mmHg. An incidental right common iliac aneurysm of 6.4 cm×6.2 cm had been detected on non-contrast computer tomography (CT) of his abdomen and pelvis at the referring hospital. An iliocaval fistula was not suspected at that time. He was started on continuous venovenous hemodiafiltration (CVVHDF) for anuric acute renal failure and fluid overload. Clinical and laboratory evidence of disseminated intravascular coagulation led to the commencement of Vitamin K and fresh frozen plasma.

An operative endovascular approach was planned in light of our patient's multiple acute comorbidities. Our patient consented to the removal of the central line with carotid angiography and endovascular balloon inflation to aid hemostasis.

Intra-operatively, with our patient in the supine position and under general anesthesia, his right common femoral artery was punctured in a retrograde manner using the standard Seldinger technique. A 5 French sheath was inserted and an attempt made at passing a 0.035 Glidewire into his abdominal aorta. The wire was noted to pass up well to the right of the midline consistent with the anatomical position of the inferior vena cava (IVC). Contrast angiography via the right femoral sheath demonstrated opacification of the right common iliac artery aneurysm as well as the IVC. It was noted that the pressure and waveform from the left internal jugular CVC displayed a mixture of arterial and venous patterns. An intra-operative diagnosis of an iliocaval fistula was made.

**Figure 1:**
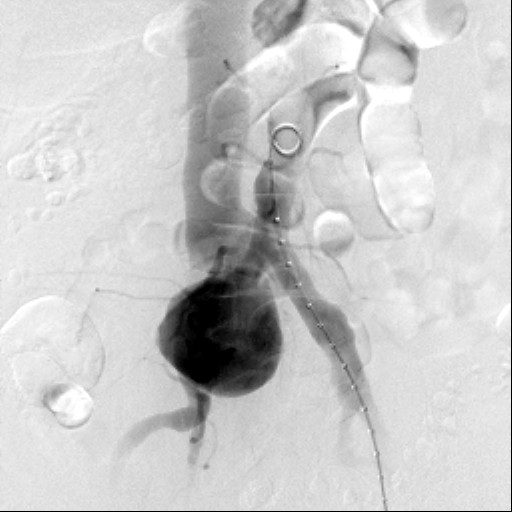
**Aortogram demonstrating a large right common iliac artery aneurysm with simultaneous contrast opacification of his inferior vena cava**.

A left common femoral artery sheath was then placed and access to his abdominal aorta was achieved. An aortogram then demonstrated the large right common iliac artery aneurysm and associated iliocaval fistula (Figure 1).

**Figure 2:**
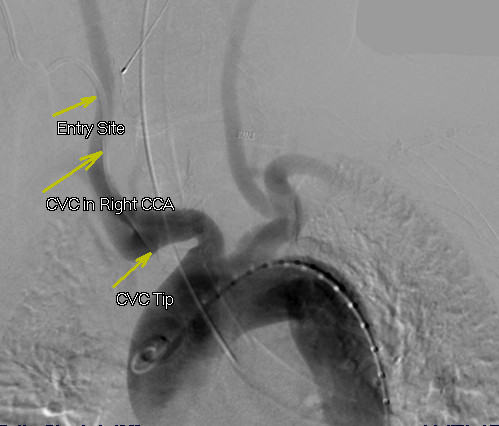
**Arch aortogram demonstrating the right-sided central venous catheter entry site into his right common carotid artery and the tip in his innominate artery**.

**Figure 3:**
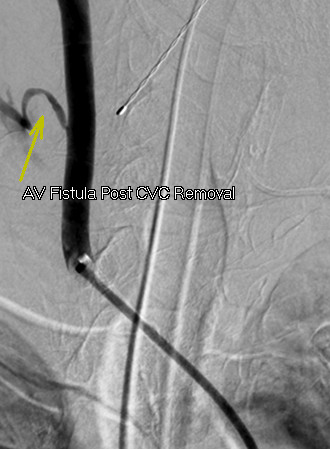
**Selective right carotid angiogram after removal of the central venous catheter, demonstrating contrast extravasation in his neck and opacification into his internal jugular vein**.

**Figure 4:**
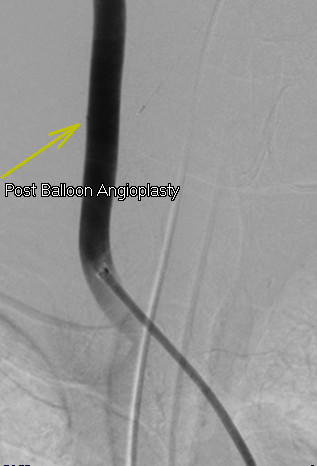
**Selective right carotid angiogram after three-minute common carotid artery balloon angioplasty, demonstrating no further contrast extravasation**.

Access into his right CCA was then achieved with a Cook Shuttle sheath (Bloomington, IN, USA). Carotid angiography demonstrated the catheter entry point into his mid-right CCA (Figure 2). The catheter was removed and angiography revealed a carotid-to-jugular arteriovenous (AV) fistula (Figure 3). An 8 mm×40 mm Abbott FoxCross (Abbott Park, IL, USA) angioplasty balloon was positioned distal to the carotid bifurcation and inflated for three minutes. Angiography then demonstrated a persistent fistula. The balloon was re-inflated for five minutes. Subsequent angiography revealed a widely patent carotid artery with no AV fistula or contrast extravasation visible (Figure 4).

Our patient remained hemodynamically unstable during surgery and, in consultation with cardiologists, the anesthetists asked that an urgent repair of the iliocaval fistula be considered during the same operation.

A calibrated aortogram was performed and bilateral Abbott Perclose devices (Abbott Park) were deployed. A 24 mm×96 mm Cook Zenith bifurcated stent graft (Bloomington) was introduced via the left iliac system. Access to his abdominal aorta was also obtained via his right femoral artery. We placed a 12 mm×107 mm TriFab Limb Extension endograft limb landing in the right external iliac artery distal to the aneurysm and iliocaval fistula. Consideration was given to coil embolization of his right internal iliac artery prior to this but it was felt not to be necessary due to the anatomy at the iliac bifurcation. A 12 mm×56 mm endograft limb was placed on the ipsilateral left side and landed in the distal left common iliac artery. There was an immediate reduction in our patient's measured central venous pressure and a classical venous waveform returned. Completion angiography demonstrated a small amount of late filling of the common iliac aneurysm via a type two endoleak but no iliocaval fistula. It was felt that, on correction of his coagulopathy, this would allow for thrombosis of the aneurysmal sac. Wires and sheaths were removed and the Perclose devices used to seal the femoral puncture sites with good effect. Our patient received four units of fresh frozen plasma, one unit of pooled platelet and eight units of cryoprecipitate.

**Figure 5:**
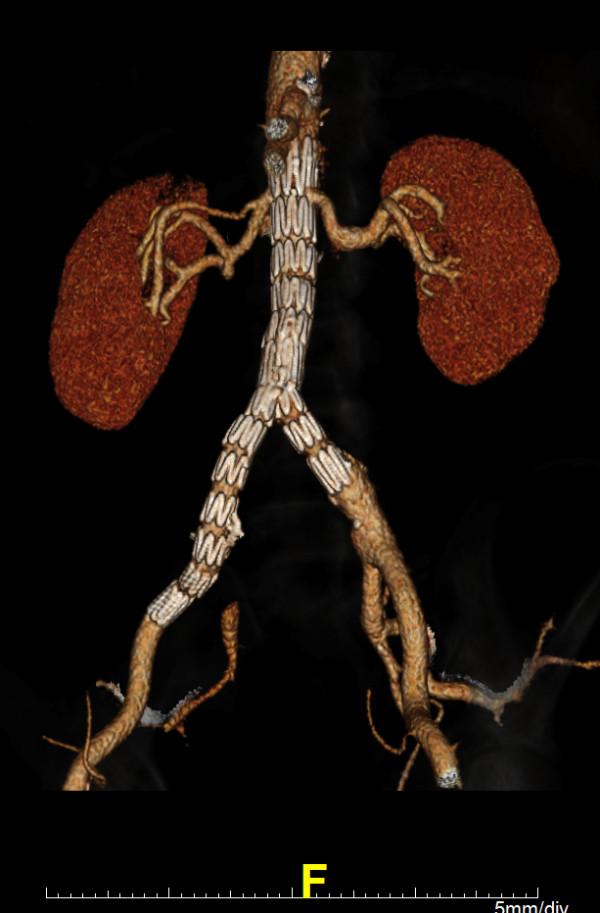
**Three-dimensional volume reconstruction of a postoperative computed tomography angiogram, demonstrating an excluded right common iliac artery aneurysm with an aortoiliac stentgraft extending into the right external iliac artery and retrograde filling of the distal right internal iliac artery**.

**Figure 6:**
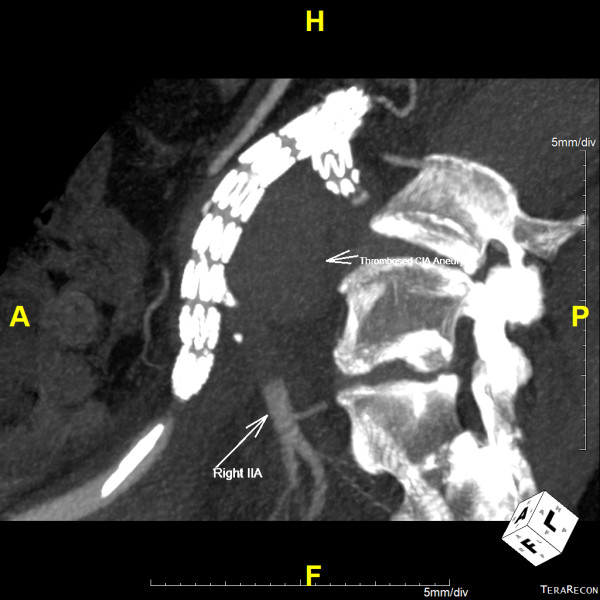
**Maximum intensity projection reconstruction of a postoperative computed tomography angiogram, demonstrating thrombus in the right common iliac artery aneurysm sac with no evidence of endoleak and retrograde filling of the right internal iliac artery stump**.

By postoperative day three, our patient's acidosis was corrected and his biochemistry and hematology parameters had improved: his Cr was 116 μmol/L, GGT was 135 IU/L, bilirubin level was 30 μmol/L, AST was 552 IU/L, Hb was 84 g/L, platelet count was 55 × 10^9^/L and INR was 1.37. His central venous pressure was normalized to 15 mmHg. He was making an adequate amount of urine and CVVHDF was ceased. Our patient was extubated successfully and a CT angiogram demonstrated a successful endovascular repair with patent endografts and no evidence of endoleak or AV fistula. Our patient was transferred back to his regional hospital after a satisfactory progress CT angiogram (Figure 5) and was discharged home three weeks later. At six-week follow-up there remained no evidence of endoleak on a progress CT angiogram (Figure 6).

## Discussion

An iliocaval fistula is a rare complication of an iliac artery aneurysm. It is an abnormal communication between the iliac artery and the IVC. The etiology can be spontaneous rupture, trauma, post-aneurysm repair or iatrogenic injury following lumbar surgery [2,3]. The incidence of aorto- or iliovenous fistula is rare and occurs in less than 1% of abdominal aortic aneurysm and 3% to 4% of ruptured abdominal aortic aneurysm [2,3].

The pathogenesis usually involves spontaneous rupture of a pre-existing aneurysm into the venous system or fistula development secondary to abdominal trauma or previous abdominal surgery, including abdominal aortic aneurysm repair and lumbar disc operation [1]. Other rare causes include mycotic aneurysm, syphilitic aneurysm and those associated with connective tissue disease, such as Ehler-Danlos syndrome [4]. In this case, two previous right femoral arterial punctures had been performed for coronary angiography. These may have contributed to the development of the iliocaval fistula. The exact etiology in this case is unknown, however the development of a pseudoaneurysm from femoral arterial catheterization with subsequent erosion into the venous system, or wire perforation into the venous system whilst manipulating a wire through an iliac artery aneurysm, both remain possibilities. Other possibilities include an atherosclerotic or degenerative iliac artery aneurysm that had spontaneously eroded into the IVC and subsequently caused high output cardiac failure. This is the primary and most common cause of development of large vessel fistulas.

The presentation of an iliocaval fistula can vary from an incidental mass to multiorgan failure. Classically, the patient presents with a triad of hypotension, pulsatile mass and heart failure [1,5]. Sometimes, the patient can present with congestive cardiac failure, leg edema and leg ischemia [5]. Gregoric *et al. *[6] first described a case of an aortocaval fistula presenting as acute renal failure. Since then, there have been only three cases [7-9] where the patient has presented with multiorgan failure. This is the second reported case of an endovascular repair of such a patient. This is the first case in which an endovascular graft was employed as the first line of treatment for such an ill patient. Schepers *et al. *[7] employed endovascular coiling as first line treatment due to their patient's condition.

Prompt diagnosis with an appropriate investigation is important in such a disease with diverse presentation. Aortic angiography used to be the investigation of choice to confirm the clinical suspicion of aortocaval or iliocaval fistula [10]. For the past two decades, CT with intravenous contrast has been used more frequently for the diagnosis of aortocaval or iliocaval fistula [8,11]. Important radiological diagnostic features include aortic or iliac artery dilatation, vena caval dilatation, increased density of the IVC similar to that of the aorta and visualization of the fistula [11].

Traditionally, fistulas associated with aortic aneurysm have been treated with open surgery [5]. However, this has been associated with high rates of morbidity and mortality, although mortality has decreased from 50% (Baker *et al. *[2]) to 20% (Brewster *et al. *[5]). Over 20 years, major morbidity has remained high. Disadvantages of open repair have included large intra-operative blood loss (mean blood loss of 4,500 mL (range 3000 mL to 8000 mL) [12] in reported cases of open repair), high intensive care unit admissions and long lengths of hospital stay. Twenty-three cases of endovascular repair of major abdominal AV fistula in the literature were reviewed, and endovascular repair had a high reported success rate of 96%, with one patient requiring open conversion, and 100% survival at a mean follow-up of nine months [1]. This case involving a patient with multiorgan failure demonstrates successful intra-operative adaptation and the immediate hemodynamic benefit possible with major AV fistula repair with no major complications.

## Conclusion

Our case demonstrates that endovascular repair of a large iliac artery aneurysm associated with a caval fistula is safe and effective and can be performed at the time of the diagnostic angiography. The presentation of an iliocaval fistula in this case was unusual which made the diagnosis difficult and unexpected at the time of surgery. The benefit of immediate repair, despite hemodynamic instability during anesthesia, is clear. Unusual iatrogenic causes of iliocaval fistula secondary to previous coronary angiograms with wire and/or catheter manipulation should be considered in patients such as ours.

## Consent

Written informed consent was obtained from the patient for publication of this manuscript and any accompanying images. A copy of the written consent is available for review by the Editor-in-Chief of this journal.

## Competing interests

The authors declare that they have no competing interests.

## Authors' contributions

YY made substantial contributions to this article in the design, literature review and write up. WM was involved in drafting the script and revising it critically. AT made a substantial contribution in acquiring the data of the patient and revising the article critically. TN made a substantial contribution in reviewing the patient's data. CF gave final approval of the version to be published. All authors read and approved the final manuscript.
